# Non-linear saturation threshold of gonadotropin dose on cumulative live birth rates in advanced-age women with polycystic ovary syndrome: a retrospective cohort study

**DOI:** 10.3389/fendo.2026.1854468

**Published:** 2026-05-20

**Authors:** Yusi Han, Chunxiao Wei, Xinhua Wang, Jianwei Zhang

**Affiliations:** 1 The First Clinical Medical College, Shandong University of Traditional Chinese Medicine, Jinan, Shandong, China; 2 Center for Reproductive Medicine and Genetics, Affiliated Hospital of Shandong University of Traditional Chinese Medicine, Jinan, Shandong, China; 3 Department of Gynecology, Jinan Jiyang District Hospital of Traditional Chinese Medicine, Jinan, Shandong, China

**Keywords:** advanced maternal age, cumulative live birth rate, dose-response relationship, endometrial thickness, gonadotropin dose, nomogram, polycystic ovary syndrome

## Abstract

**Background:**

Polycystic ovary syndrome (PCOS) is a leading cause of anovulatory infertility. Despite a high antral follicle count, advanced-age PCOS patients experience a marked decline in cumulative live birth rates (CLBR) after assisted reproductive technology. The dose-response relationship between total gonadotropin (Gn) dose and CLBR in this population remains poorly defined. This study aimed to quantify the non-linear threshold effect of total Gn dose, characterize the endometrial thickness (EMT) dose-response relationship, and develop a predictive nomogram.

**Methods:**

This retrospective cohort study analyzed 2449 PCOS patients stratified by age quartiles. Univariate and multivariable logistic regression identified predictors of CLBR for the entire cohort and the advanced-age subgroup (≥35 years). For the advanced-age group, restricted cubic splines (RCS) evaluated non-linear associations between total Gn dose and CLBR; piecewise linear regression identified saturation thresholds. RCS and conditional marginal effects analyzed EMT-CLBR relationships. A nomogram was constructed from multivariable predictors and validated internally with 1000 bootstrap resamples. Model performance was assessed via AUC-ROC, calibration curves, Hosmer-Lemeshow test, and decision curve analysis.

**Results:**

CLBR was significantly lower in the advanced-age group (44.40%) than in younger cohorts (63.90%–69.40%, *P* < 0.001). In the advanced-age subgroup, total Gn dose and EMT independently predicted CLBR. RCS confirmed a non-linear dose-response relationship (*P* = 0.019), with saturation at 1, 600 IU. Below this threshold, each 100-IU increase was associated with higher odds of CLBR (aOR: 1.059, *P* = 0.034); above it, no significant association was observed (*P* = 0.624). EMT showed a stable positive linear association with CLBR (aOR: 1.088, *P* = 0.016), with predicted CLBR rising from 32.5% at 6 mm to 66.1% at 22 mm. The nomogram incorporating age, EMT, transfer type, AFC, and Gn dose demonstrated moderate discrimination (AUC = 0.74) and adequate calibration (*P* = 0.108).

**Conclusions:**

CLBR was substantially lower in advanced-age PCOS patients. A non-linear association between total Gn dose and CLBR was observed, with a plateau at approximately 1, 600 IU, although this finding may reflect confounding by indication. EMT showed a stable positive linear association with CLBR. These findings suggest that moderate stimulation with attention to endometrial preparation may warrant consideration in this population.

## Introduction

Polycystic Ovary Syndrome (PCOS) affects 11%–13% of women of reproductive age worldwide (1, 2) and is the underlying cause in 80%–90% of anovulatory infertility cases (3, 4). PCOS is characterized by hyperandrogenism, ovulatory dysfunction, and insulin resistance, which collectively impair follicular development and endometrial receptivity (5). Given the contemporary trend of delayed childbearing, an increasing cohort of PCOS patients is seeking Assisted Reproductive Technology (ART) at age 35 or older (6). While PCOS is typically associated with a high Antral Follicle Count (AFC), this quantitative ovarian reserve advantage may be offset by age-related declines in oocyte quality and the adverse effects of the PCOS metabolic milieu on the endometrium (7–9). Consequently, a high AFC in older PCOS patients should not be viewed as a definitive indicator of favorable reproductive outcomes (10, 11). In clinical practice, the total Gonadotropin (Gn) dose and Endometrial Thickness (EMT) are commonly used to guide stimulation protocols and embryo transfer decisions. In younger PCOS patients, the primary complication associated with high Gn doses is ovarian hyperstimulation syndrome. Conversely, in patients of advanced reproductive age, both insufficient and excessive stimulation may compromise oocyte competence and endometrial receptivity (12–14). Most prior studies have examined Gn dose effects under the assumption of a linear dose-response relationship, and no established dose threshold exists for advanced-age PCOS patients (15, 16). Similarly, the independent contribution of EMT to cumulative live birth rate (CLBR) in this population remains poorly quantified. This study hypothesized that the association between total Gn dose and CLBR in advanced-age patients with polycystic ovary syndrome (PCOS) is non-linear, characterized by a threshold beyond which further dose escalation yields no additional benefit. To validate this hypothesis, an age-stratified analysis of CLBR was performed. Furthermore, restricted cubic splines and piecewise regression were applied to identify the Gn dose threshold, the association between EMT and CLBR was evaluated, and a predictive nomogram was constructed.

## Materials and methods

### Study design and participants

**Figure 1:**
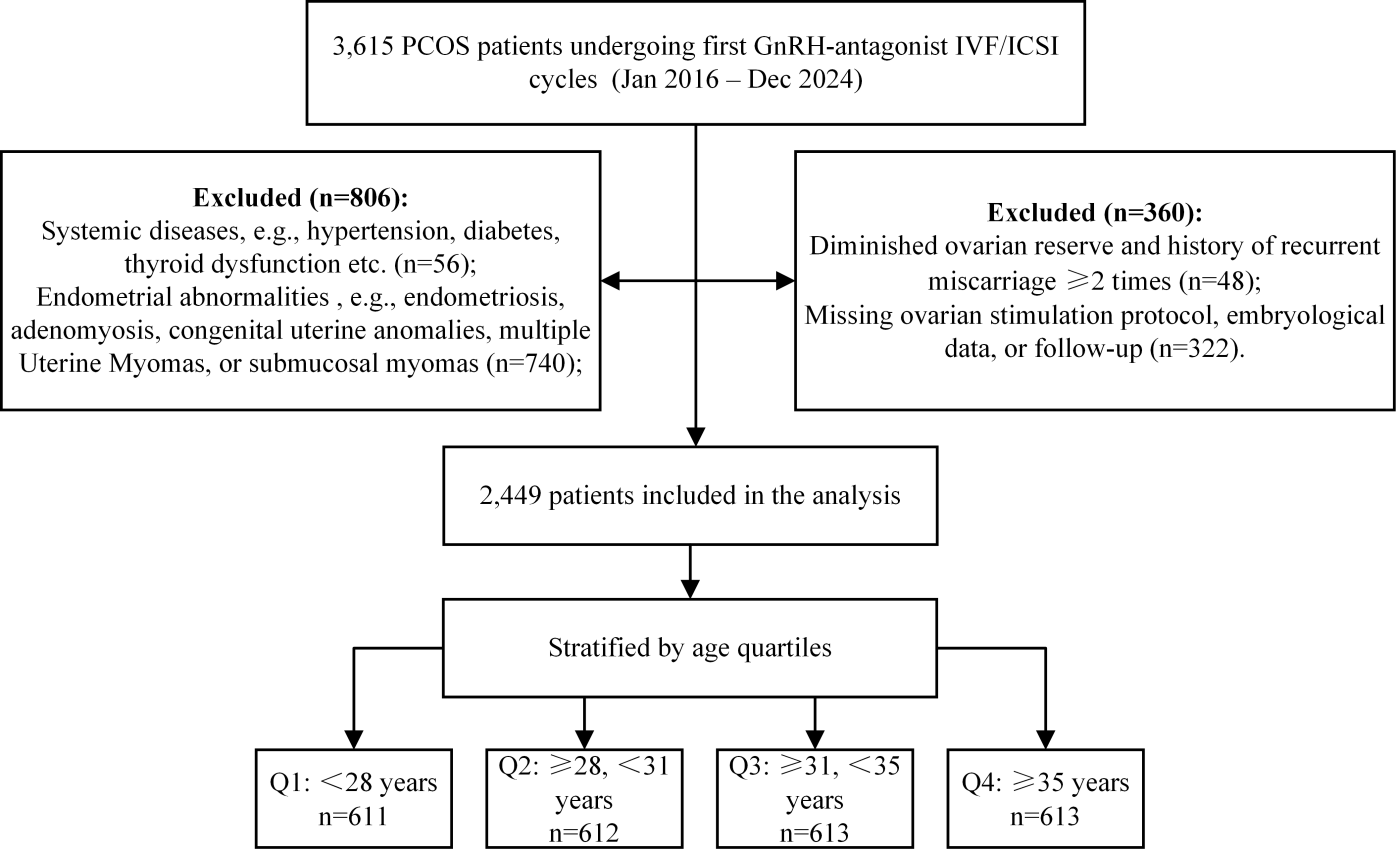
Flow chart showing the identification of the study population.

This retrospective cohort study was conducted at the Affiliated Hospital of Shandong University of Traditional Chinese Medicine. This study identified 3, 615 PCOS patients who underwent their first IVF/ICSI cycle using a gonadotropin-releasing hormone antagonist (GnRH-ant) regimen between January 2016 and December 2024. PCOS was diagnosed according to the 2004 Rotterdam criteria (17), requiring at least two of the following: 1) oligo- or anovulation; 2) clinical or biochemical hyperandrogenism; and 3) polycystic ovarian morphology on ultrasound. Exclusion criteria included: 1) endometriosis, submucosal uterine fibroids, or uterine malformations; 2) other known endocrine or metabolic disorders; 3) diminished ovarian reserve; 4) a history of recurrent miscarriage (≥2 cases); and 5) incomplete medical records. After screening 2,449 patients were included in the final analysis (**Figure 1**). To evaluate the impact of age, the cohort was stratified into four groups based on age quartiles. The study endpoint was the first live birth or the depletion of all embryos derived from the initial stimulation cycle.

### Ethical approval and data collection

The study protocol was approved by the Ethics Committee of the Affiliated Hospital of Shandong University of Traditional Chinese Medicine (No. 2026-014-01-KY). As this was a retrospective analysis of pre-existing anonymized clinical records, the ethics committee granted a waiver of study-specific informed consent, in accordance with institutional policy for retrospective studies using de-identified data. All patients had provided written general consent for the use of their clinical data for research purposes at the time of their IVF treatment. All demographic and clinical information was extracted from the center’s electronic medical record system. Baseline characteriztics included age, body mass index (BMI), infertility type and duration, miscarriage history, and AFC. Basal hormonal profiles comprised follicle-stimulating hormone (FSH), luteinizing hormone (LH), estradiol (E_2_), progesterone (P), and testosterone (T). Treatment-specific variables included the total Gn dose, stimulation duration, number of retrieved oocytes, and fertilization method (IVF or ICSI). Additionally, we recorded the number of available embryos, EMT, transfer type (fresh or frozen), and embryo stage (cleavage-stage or blastocyst).

### Ovarian stimulation protocols

All included patients received a standardized GnRH-ant regimen. Controlled ovarian stimulation was initiated on approximately day 3 of menstruation or withdrawal bleeding using recombinant follicle-stimulating hormone (rFSH; Gonal-f, Merck) at a starting dose of 150–300 IU/day. Follicular development was monitored by transvaginal ultrasound and by measuring E_2_, LH, and P levels, with individualized adjustments to the Gn dose. Daily subcutaneous administration of cetrorelix acetate (Cetrotide, Merck Serono, 0.25 mg/day) was initiated on day 6 of Gn stimulation to prevent a premature LH surge. When at least one dominant follicle reached a diameter of ≥18 mm, human chorionic gonadotropin (hCG) was administered to trigger final oocyte maturation. Oocyte retrieval was performed 36 hours later.

### Embryo grading and quality evaluation

Embryo grading was performed according to the Istanbul consensus (18) and Gardner grading system (19). On day 3, cleavage-stage embryos were evaluated based on blastomere number, symmetry, fragmentation, and cytoplasmic granularity. Morphological scores categorized embryos as poor-quality (<6, excluded), viable (≥6), or high-quality (≥7). Blastocysts were assessed by blastocoel expansion and the morphology of the inner cell mass and trophectoderm. High-quality embryos were prioritized for both fresh and frozen-thawed embryo transfer (ET/FET). Luteal phase support with progesterone was initiated after transfer and continued until the 10th week of gestation.

### Outcome measurement

The primary outcome was the CLBR. The secondary outcome was the cumulative clinical pregnancy rate (CCPR). CLBR was defined as the first live birth achieved using all fresh and frozen embryos derived from a single ovarian stimulation cycle. Similarly, CCPR was defined as the first clinical pregnancy resulting from all embryo transfers within one complete cycle. CCPR was reported descriptively; formal regression analysis focused on CLBR as the primary outcome. To ensure data independence and avoid confounding effects from repeated treatments, only the first stimulation cycle for each patient was analyzed (20).

### Statistical analysis

Statistical analyses were performed using SPSS (version 27.0) and R (version 4.4.2). Continuous variables were assessed for normality using the Shapiro-Wilk test. As all continuous variables showed non-normal distributions, they are presented as medians (interquartile ranges) and compared using the Kruskal-Wallis test with Bonferroni-corrected pairwise comparisons. Categorical variables were reported as frequencies (%) and were compared using Pearson’s χ^2^ or Fisher’s exact tests. All tests were two-tailed, with significance set at *P <*0.05. Variables with missing data (<5% for all included variables) were handled via multiple imputation by chained equations (MICE), with five imputed datasets generated and estimates pooled according to Rubin’s rules. Univariate and multivariable logistic regression identified independent predictors of CLBR. Multicollinearity was evaluated using the variance inflation factor (VIF), with VIF <5 considered acceptable. Results were reported as adjusted odds ratios (aOR) with 95% confidence intervals (CI). To evaluate non-linear associations between total Gn dose, EMT, and CLBR, we employed restricted cubic splines (RCS) with four knots (5th, 35th, 65th, and 95th percentiles). RCS was used because it can model non-linear dose-response relationships without assuming a constant dose-effect slope, and it provides smooth, interpretable curves with sufficient power to detect departures from linearity. Threshold effects were identified via piecewise linear regression, using bootstrap resampling (1000 iterations) to estimate 95% CIs for breakpoints. Conditional marginal effects were calculated to quantify CLBR probability changes per 1-mm increase in EMT. A nomogram was constructed based on key predictors from the multivariable model. Internal validation used 1000 bootstrap resamples. Model discrimination was assessed by the area under the receiver operating characteriztic (ROC) curve (AUC), while goodness-of-fit was evaluated using calibration curves and the Hosmer-Lemeshow test. Clinical utility was determined through decision curve analysis (DCA).

## Results

### Baseline characteriztics by age quartile

A total of 2449 patients were included and stratified into four groups based on age quartiles, with resulting cutoff values of 28, 31, and 35 years. This yielded four subgroups: Q1 (<28 years, n = 611), Q2 (28–30 years, n = 612), Q3 (31–34 years, n = 613), and Q4 (≥35 years, n = 613). Baseline characteriztics across groups are summarized in **Table 1**. The advanced-age group (Q4) exhibited a significantly higher median BMI than the Q2 group (*P* < 0.05). Both the proportion of primary infertility and the duration of infertility increased progressively with age (both *P* < 0.001). Regarding basal hormone profiles, FSH levels rose significantly with advancing age (*P* < 0.001), while LH and P levels were significantly lower in the Q4 group compared to all other groups (all *P* < 0.05). Indicators of ovarian reserve began to decline significantly in Q3, reaching their lowest values in Q4 (*P* < 0.001). Regarding ovarian stimulation, the total Gn dose was significantly higher in the Q4 group than in all other groups (*P* < 0.001). Both the number of oocytes retrieved and the number of viable embryos decreased as age increased (*P* < 0.001). Furthermore, EMT was significantly thinner in the Q4 group compared to the younger cohorts (*P* < 0.001). Patients in the Q4 group were more likely to undergo fresh embryo transfers (*P* < 0.001). No statistically significant differences were observed among the groups regarding E_2_, T, ovarian volume, duration of Gn stimulation, fertilization technology, or embryo type.

**Table 1:** Baseline characteriztics by age quartile.

Variables	Q1 <28 years	Q2 ≥28, <31 years	Q3 ≥31, <35 years	Q4 ≥35 years	*P*-value
Number of cycles	n = 611	n = 612	n = 613	n = 613	*-*
Age (y)	26 (25, 27)	30 (29, 31)^a^	33 (32, 33)^a,b^	36 (35, 38)^a,b,c^	<0.001^1^
BMI (kg/m²)	24.40 (21.30, 28.20)	24.15 (21.50, 27.30)	24.00 (21.40, 26.95)	24.50 (22.30, 27.35) ^‡^	0.042^1^
Type of infertility, n (%)					
Primary	179 (29.30)	228 (37.30)^a^	303 (49.40)^a,b^	431 (70.30)^a,b,c^	<0.001^2^
Secondary	432 (70.70)	384 (62.70)^a^	310 (50.60)^a,b^	182 (29.70)^a,b,c^	<0.001^2^
Duration of infertility (y)	2 (1, 3)	3 (2, 4)^a^	3 (2, 5)^a,b^	3 (2, 6)^a,‡^	<0.001^1^
Number of miscarriages, n (%)					0.367^2^
1	5 (0.80)	7 (1.10)	5 (0.80)	11 (1.80)	
0	606 (99.20)	605 (98.90)	608 (99.20)	602 (98.20)	
Baseline FSH (IU/L)	6.00 (5.01, 7.14)	6.17 (5.21, 7.22)	6.26 (5.26, 7.56)^a^	6.43 (5.47, 7.66)^a,b^	<0.001^1^
Baseline LH (IU/L)	5.09 (3.35, 7.93)	5.04 (3.68, 7.52)	5.20 (3.72, 7.33)	4.51 (3.39, 6.46)^a,b,c^	<0.001^1^
Baseline E_2_ (pg/mL)	35.59 (27.24, 46.42)	36.12 (27.76, 47.76)	35.84 (27.87, 48.35)	35.39 (27.77, 45.30)	0.752^1^
Baseline P (ng/mL)	0.49 (0.31, 0.72)	0.48 (0.30, 0.71)	0.45 (0.27, 0.66)^a^	0.40 (0.24, 0.62)^a,b,§^	<0.001^1^
Baseline T (ng/mL)	3.01 (1.81, 4.04)	3.13 (1.86, 4.05)	2.85 (1.69, 4.09)	2.89 (1.77, 4.17)	0.877^1^
Ovarian volume (cm^3^)	5.16 (4.31, 6.31)	5.02 (4.32, 6.51)	5.13 (4.34, 6.45)	5.19 (4.34, 6.89)	0.619^1^
AFC	22.00 (20.00, 24.00)	22.00 (20.00, 24.00)	20.00 (20.00, 24.00)^a,b^	20.00 (19.00, 22.00)^a,b,c^	<0.001^1^
Total Gn dose (IU)	1800.00 (1475.00, 2250.00)	1837.50 (1520.00, 2243.75)	1900.00 (1, 600.00, 2250.00)^a^	2025.00 (1725.00, 2475.00)^a,b,c^	<0.001^1^
Duration of Gn stimulation (d)	9 (8, 10)	9 (8, 10)	9 (8, 10)	9 (8, 10)	0.346^1^
No. of oocytes retrieved	16 (11, 21)	15 (10, 20)^a^	13 (9, 19)^a,b^	11 (7, 16)^a,b,c^	<0.001^1^
Fertilization technology, n (%)					
IVF	449 (73.50)	479 (78.30)	465 (75.90)	460 (75.00)	0.265^2^
ICSI	162 (26.50)	133 (21.70)	148 (24.10)	153 (25.00)	
No. of available embryos	2 (2, 4)	2 (2, 4)	2 (2, 4)	2 (2, 4)^†,b^	0.007^1^
EMT (mm)	12.00 (10.50, 13.70)	12.10 (10.60, 13.70)	12.00 (10.25, 13.55)	11.50 (10.00, 13.25)^a,b,c^	<0.001^1^
No. of embryos transferred	2 (1, 2)	2 (1, 2)	2 (1, 3)	2 (1, 3)	0.158^1^
Transfer type, n (%)					<0.001^2^
ET	176 (28.80)	184 (30.10)	210 (34.30)^a^	274 (44.70)^a,b,c^	
FET	435 (71.20)	428 (69.90)	403 (65.70)^a^	339 (55.30)^a,b,c^	
Embryo type, n (%)					0.899^2^
Cleavage embryo	570 (93.30)	576 (94.10)	573 (93.50)	577 (94.10)	
Blastocyst	41 (6.70)	36 (5.90)	40 (6.50)	36 (5.90)	

Values are numbers (percentages) or median (interquartile range). Statistical tests: ¹Kruskal-Wallis test with Bonferroni *post-hoc* correction; ²Chi-square test with Bonferroni *post-hoc* correction. Superscript letters denote statistically significant pairwise differences at *P* < 0.001: ^a^ vs. Q1; ^b^ vs. Q2; ^c^ vs. Q3. Symbols denote statistically significant pairwise differences at *P* < 0.05: † vs. Q1; ‡ vs. Q2; § vs. Q3.

### Pregnancy outcomes by age quartiles

Pregnancy outcomes deteriorated significantly in the advanced-age group (Q4) compared with younger cohorts (**Table 2**). Compared to cohorts Q1–Q3, the Q4 group exhibited markedly lower CCPR (48.50% vs. 70.70%, 69.30%, and 66.40%, respectively; *P* < 0.001) and CLBR (44.40% vs. 69.40%, 67.80%, and 63.90%, respectively; *P* < 0.001).

**Table 2:** Pregnancy outcomes by age quartiles.

Pregnancy outcomes	Q1	Q2	Q3	Q4	*P*-Value
Number of cycles	n = 611	n = 612	n = 613	n = 613	*-*
CCPR, n (%)	432 (70.70)	424 (69.30)	407 (66.40)	297 (48.50)^a,b,c^	<0.001^1^
CLBR, n (%)	424 (69.40)	415 (67.80)	392 (63.90)^a^	272 (44.40)^a,b,c^	<0.001^1^

Values are numbers (percentages). Statistical tests: ¹ Chi-square test with Bonferroni *post-hoc* correction. Superscript letters denote statistically significant pairwise differences at *P* < 0.001:^a^ vs. Q1; ^b^ vs. Q2; ^c^ vs. Q3.

### Univariate and multivariable analysis of factors associated with CLBR in the entire cohort

Univariate logistic regression identified several factors significantly associated with CLBR (**Table 3**). Age, BMI, infertility duration, and baseline FSH were inversely associated with CLBR, while baseline LH, P, AFC, the number of oocytes retrieved, the number of available embryos, EMT, and FET showed positive associations (all *P* < 0.05). Variables were selected for the multivariable model based on clinical relevance and a univariate threshold of *P* < 0.10. To avoid over-adjustment, the number of oocytes retrieved and available embryos were excluded as potential mediating variables. Multicollinearity was negligible, with all VIFs below 2.0. Multivariable analysis revealed that, compared to the Q1 group, the advanced-age group (Q4) had a significantly lower CLBR (aOR: 0.442, 95% CI 0.344-0.566, *P* < 0.001). In contrast, no significant differences were observed for the Q2 or Q3 groups compared to Q1 (both *P >*0.05). Other independent risk factors for decreased CLBR included a longer duration of infertility (aOR: 0.943, *P* < 0.001), increased total Gn dose (aOR: 0.979 per 100 IU; *P* = 0.002), and the use of IVF (aOR: 0.744; *P* = 0.004). Conversely, higher baseline LH (aOR: 1.030; *P* = 0.027), higher baseline P (aOR: 1.402; *P* = 0.003), increased EMT (aOR: 1.055; *P* = 0.003), and FET cycles (aOR: 1.355; *P* = 0.001) were independently associated with a higher probability of CLBR.

**Table 3:** Univariate and multivariable analysis of factors associated with CLBR in the entire cohort.

Variables	Univariate regression		Multivariable regression	
	Unadjusted OR (95% CI)	*P*-Value	aOR (95% CI)	*P*-Value ^a^
Group				
Q2	0.929 (0.730-1.183)	0.551	0.988 (0.773-1.263)	0.924
Q3	0.782 (0.616-0.993)	0.043	0.881 (0.689-1.128)	0.315
Q4	0.352 (0.278-0.445)	<0.001	0.442 (0.344-0.566)	<0.001
Q1	Ref	Ref	Ref	Ref
BMI (kg/m²)	0.981 (0.962-0.999)	0.044	0.999 (0.976-1.022)	0.910
Type of infertility				
Secondary	1.111 (0.944-1.308)	0.204		
Primary	Ref	Ref		
Duration of infertility (y)	0.921 (0.891-0.952)	<0.001	0.943 (0.910-0.978)	<0.001
Baseline FSH (IU/L)	0.95 (0.908-0.994)	0.027	0.966 (0.919-1.015)	0.173
Baseline LH (IU/L)	1.038 (1.014-1.064)	0.002	1.030 (1.003-1.058)	0.027
Baseline E_2_ (pg/mL)	1.000 (0.997-1.002)	0.881		
Baseline P (ng/mL)	1.544 (1.243-1.919)	<0.001	1.402 (1.123-1.750)	0.003
AFC	1.019 (1.005-1.033)	0.008	1.002 (0.987-1.017)	0.769
Total Gn dose (100 IU)	0.969 (0.957-0.98)	<0.001	0.979 (0.966-0.992)	0.002
Duration of Gn stimulation (d)	0.964 (0.924-1.006)	0.096		
No. of oocytes retrieved	1.045 (1.033-1.057)	<0.001		
Fertilization technology				
IVF	0.772 (0.636-0.936)	0.009	0.744 (0.608-0.911)	0.004
ICSI	Ref	Ref	Ref	Ref
No. of available embryos	1.115 (1.065-1.168)	<0.001		
EMT (mm)	1.067 (1.03-1.104)	<0.001	1.055 (1.018-1.094)	0.003
Transfer type				
FET	1.609 (1.357-1.907)	<0.001	1.355 (1.124-1.633)	0.001
ET	Ref	Ref	Ref	Ref
Embryo type				
Blastocyst	1.165 (0.827-1.64)	0.382		
Cleavage embryo	Ref	Ref		

a Adjusted for age group, BMI, duration of infertility, baseline FSH, baseline LH, baseline P, AFC, Gn dose, fertilization technology, EMT, and transfer type.

### Univariate and multivariable analysis of factors associated with CLBR in advanced-age PCOS patients

To identify specific predictors of CLBR for advanced-age PCOS patients (≥35 years), we performed a multivariable analysis of the Q4 subgroup (n = 613; **Table 4**). Univariate analysis indicated that CLBR was inversely associated with age, secondary infertility, and total Gn dose. Conversely, AFC, number of oocytes retrieved, number of available embryos, EMT, and FET cycles were positively associated with CLBR. Variables with *P* < 0.10 in the univariate analysis or those of high clinical relevance were included in the multivariable model. To avoid over-adjustment, the number of oocytes retrieved and available embryos were excluded as potential mediators. Multicollinearity was not significant (VIF <2).

**Table 4:** Univariate and multivariable analysis of factors associated with CLBR in advanced-age PCOS patients.

Variables	Univariate regression		Multivariable regression	
	Unadjusted OR (95% CI)	*P*-Value	aOR (95% CI)	*P*-Value ^a^
Age (y)	0.937 (0.879-0.998)	0.045	0.949 (0.887-1.017)	0.137
BMI (kg/m²)	0.990 (0.951-1.030)	0.615		
Type of infertility				
Secondary	0.642 (0.450-0.917)	0.015	0.626 (0.421-0.930)	0.021
Primary	Ref	Ref	Ref	Ref
Duration of infertility (y)	0.957 (0.913-1.003)	0.069	0.977 (0.926-1.031)	0.394
Baseline FSH (IU/L)	0.938 (0.860-1.024)	0.153		
Baseline LH (IU/L)	1.035 (0.985-1.088)	0.172		
Baseline E_2_ (pg/mL)	0.997 (0.992-1.003)	0.311		
Baseline P (ng/mL)	1.520 (0.994-2.325)	0.064	1.437 (0.923-2.236)	0.108
AFC	1.040 (1.008-1.072)	0.012	1.008 (0.973-1.044)	0.141
Total Gn dose (100 IU)	0.975 (0.953-0.997)	0.025	0.975 (0.952-0.998)	0.026
Duration of Gn stimulation (d)	1.005 (0.927-1.089)	0.909		
No. of oocytes retrieved	1.046 (1.022-1.071)	<0.001		
Fertilization technology				
IVF	0.774 (0.514-1.165)	0.22		
ICSI	Ref	Ref		
No. of available embryos	1.097 (1.002-1.202)	0.046		
EMT (mm)	1.084 (1.019-1.154)	0.011	1.088 (1.016-1.165)	0.016
Transfer type				
FET	1.563 (1.131-2.160)	0.007	1.322 (0.927-1.884)	0.123
ET	Ref	Ref	Ref	Ref
Embryo type				
Blastocyst	1.130 (0.575-2.218)	0.723		
Cleavage embryo	Ref	Ref		

a Adjusted for age, type of infertility, duration of infertility, baseline P, AFC, Gn dose, EMT, and transfer type.

The final multivariable model identified three independent predictors. EMT was the strongest positive predictor: each 1-mm increase in thickness was associated with 8.8% higher odds of achieving a cumulative live birth (aOR: 1.088; 95% CI: 1.016–1.165; *P* = 0.016). In contrast, total Gn dose showed an independent negative association, with each 100-IU increase corresponding to 2.5% lower odds of achieving a cumulative live birth (aOR: 0.975; 95% CI: 0.952–0.998; *P* = 0.026). Additionally, patients with secondary infertility had 37.4% lower odds of achieving a cumulative live birth compared to those with primary infertility (aOR: 0.626; 95% CI: 0.421–0.930; *P* = 0.021).

### Non-linear relationship and threshold effect of total Gn dose on CLBR in advanced-age PCOS patients

**Figure 2:**
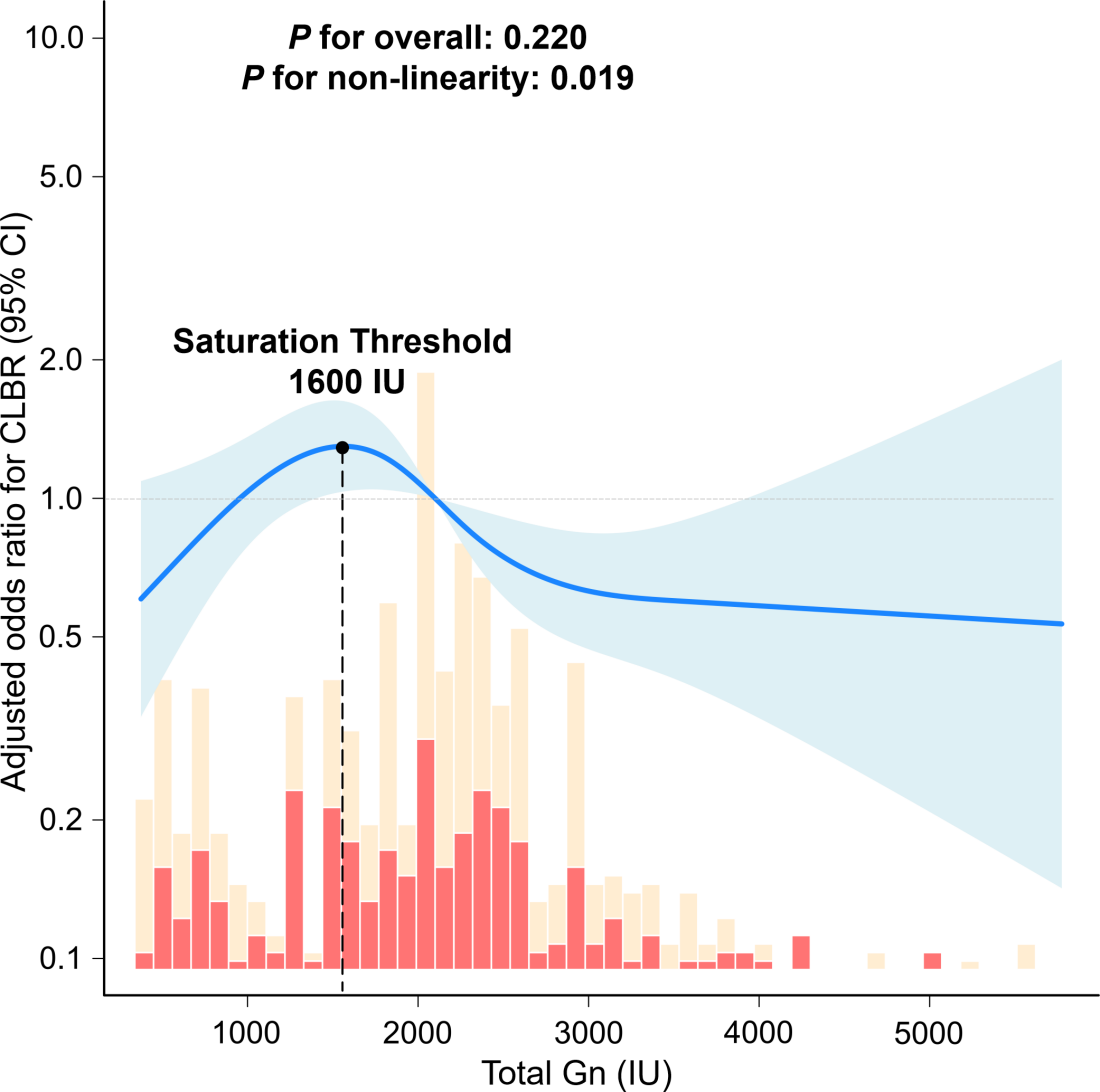
RCS curve fitting illustrating the non-linear association between total Gn dose and CLBR. The solid blue line represents the aOR of CLBR as a function of total Gn dose, with the shaded area indicating the 95% CI.

In the multivariable logistic regression, total Gn dose was an independent predictor of CLBR, with each 100-IU increment associated with a significantly lower probability of live birth (aOR: 0.975, 95% CI 0.952-0.998, *P* = 0.026). To further characterize this dose-response relationship, this study employed RCS regression adjusted for the same covariates. The likelihood ratio test confirmed a significant non-linear association (*P* for non-linearity = 0.019, **Figure 2**). A two-piecewise linear regression model determined an optimal threshold of approximately 1, 600 IU for the total Gn dose. Below this threshold (<1, 600 IU), each additional 100 IU was associated with 5.9% higher odds of achieving a cumulative live birth (aOR: 1.059; 95% CI: 1.005-1.117; P = 0.034). However, beyond 1, 600 IU, no statistically significant association was observed between additional Gn dose and CLBR (aOR: 0.983; 95% CI: 0.955–1.012; *P* = 0.624; **Table 5**).

**Table 5:** Saturation effect analysis of total Gn dose on CLBR in advanced-age PCOS patients.

Total Gn dose (100 IU)	aOR (95% CI)	*P*-value
One-line linear regression model	0.975 (0.952-0.998)	0.026
Two-piecewise linear regression model		
<1, 600	1.059 (1.005-1.117)	0.034
≥1, 600	0.983 (0.955-1.012)	0.624
Likelihood Ratio test ^a^		<0.001

a The Likelihood Ratio Test compared the goodness-of-fit of the two-piecewise linear regression model against the one-line linear regression model.

### Linear association between EMT and CLBR in advanced-age patients

**Figure 3:**
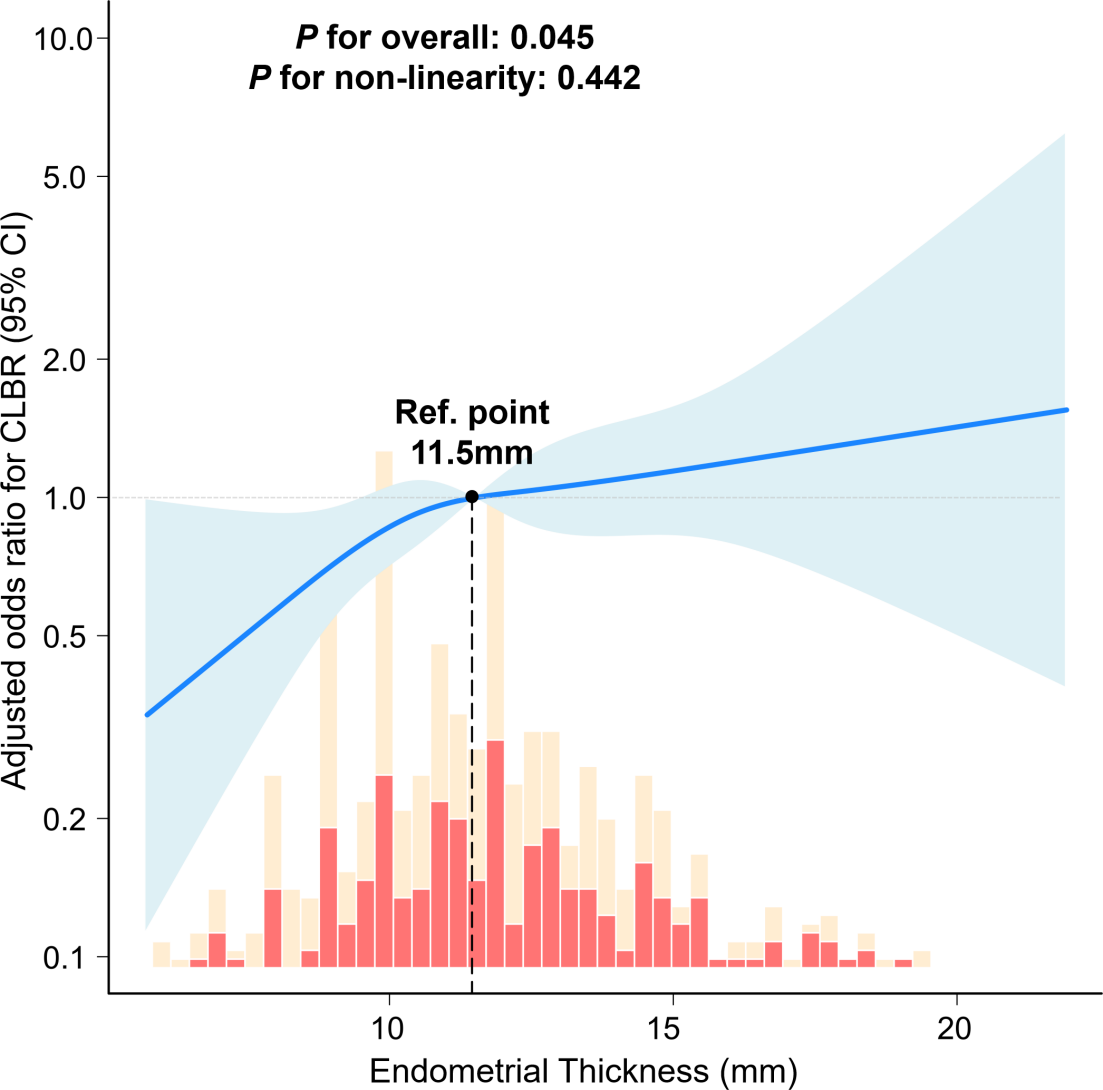
Association between EMT and CLBR in advanced-age PCOS patients.

**Figure 4:**
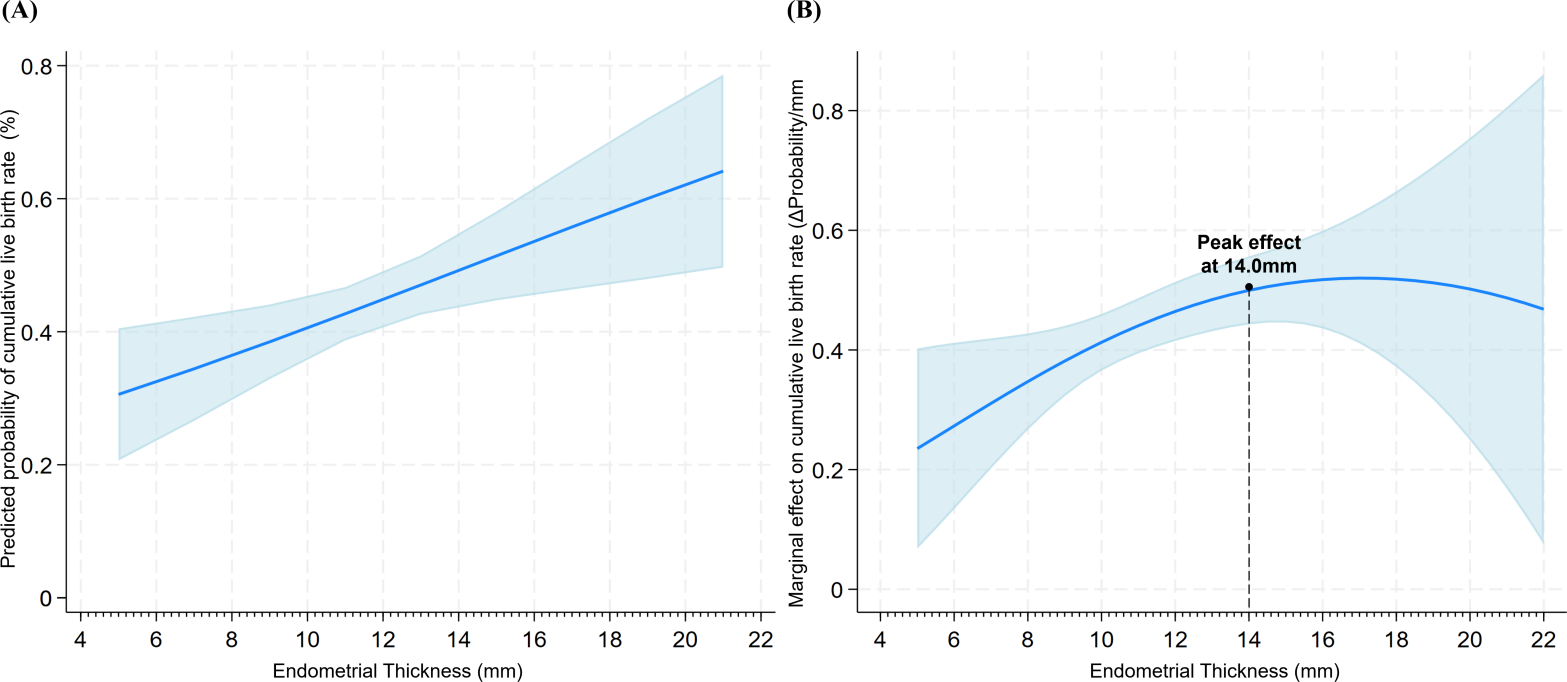
Predicted probability and conditional marginal effects of EMT on CLBR. **(A)** Predicted probability of CLBR (solid line) with 95% CIs (shaded area) as a function of EMT in advanced-age PCOS patients. **(B)** Conditional marginal effects representing the absolute change in CLBR probability (percentage points) per 1-mm increase in EMT. Shaded regions indicate 95% CIs calculated via the delta method.

This study further evaluated the association between EMT and CLBR using the same adjusted multivariable model. RCS analysis yielded a P-value for non-linearity of 0.442, indicating no significant departure from linearity (**Figure 3**). Conditional marginal effects analysis demonstrated that as EMT increased from 6 mm to 22 mm, the predicted CLBR rose progressively from 32.5% to 66.1% (**Figure 4A**; **Table 6**). Notably, the predicted CLBR surpassed 40% once EMT reached 10 mm. The marginal effect peaked at 14 mm, with a gain of 2.19 percentage points per mm (95% CI: 0.5–3.8 percentage points per mm; *P <*0.01; **Figure 4B**). All marginal effects remained statistically significant across the measured range. In summary, EMT exhibited a stable positive linear association with CLBR across the observed range in advanced-age PCOS patients.

**Table 6:** Predicted probabilities and conditional marginal effects of EMT on CLBR in advanced-age PCOS patients.

EMT (mm)	Predicted probability (%, 95% CI) ^a^	Conditional marginal effect (percentage point per mm, 95% CI) ^b^	*P*-value ^c^
6	32.5 (23.6-41.4)	1.93 (0.7-3.1)	0.003
8	36.4 (29.7-43.1)	2.03 (0.6-3.4)	0.006
10	40.6 (35.9-45.2)	2.11 (0.6-3.6)	0.008
12	44.9 (40.9-48.8)	2.16 (0.5-3.8)	0.009
14	49.2 (43.8-54.6)	2.19 (0.5-3.8)	0.009
16	53.6 (45.6-61.6)	2.18 (0.6-3.8)	0.008
18	57.9 (47.1-68.7)	2.14 (0.7-3.6)	0.006
20	62.1 (48.8-75.4)	2.06 (0.8-3.3)	0.003
22	66.1 (50.6-81.7)	1.97 (0.9-3.0)	<0.001

a Predicted probabilities were derived from the multivariable logistic regression model, adjusted for age, type of infertility, duration of infertility, baseline P, AFC, total Gn dose and transfer type. 95% CI was calculated by the delta method.

b Conditional marginal effects represent the absolute change in CLBR probability (in percentage points) per 1 mm increase in EMT at the specified thickness. All Wald test *P* values <0.01. 95% CI was calculated by the delta method.

c Wald test *P*-value for the conditional marginal effect at the specified EMT value.

### Construction and validation of a prediction model for CLBR in advanced-age PCOS patients

#### Establishment of the nomogram model

**Figure 5:**
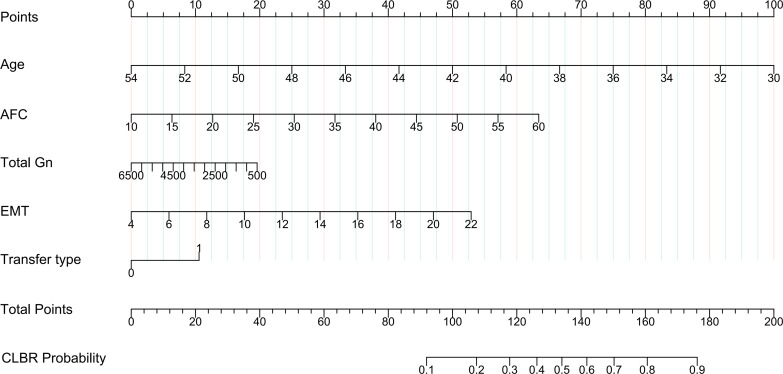
Nomogram for predicting the individualized probability of CLBR in advanced-age PCOS patients.

This study developed a nomogram to predict the probability of CLBR in advanced-age PCOS patients (**Figure 5**). The model incorporated five clinically relevant variables identified from the multivariable analysis. Although age, AFC, and transfer type did not reach statistical significance in this subgroup, they were retained based on their established clinical importance in reproductive medicine and their contribution to overall model performance. Secondary infertility, although statistically significant in the subgroup analysis, was excluded from the nomogram because it is a non-modifiable characteriztic that cannot guide treatment planning. In this scoring system, each variable is assigned a point value; the cumulative score is then mapped to estimate the individualized probability of achieving a live birth. Internal validation was performed using 1000 bootstrap resamples to ensure model stability.

#### Evaluation of the nomogram model

**Figure 6:**
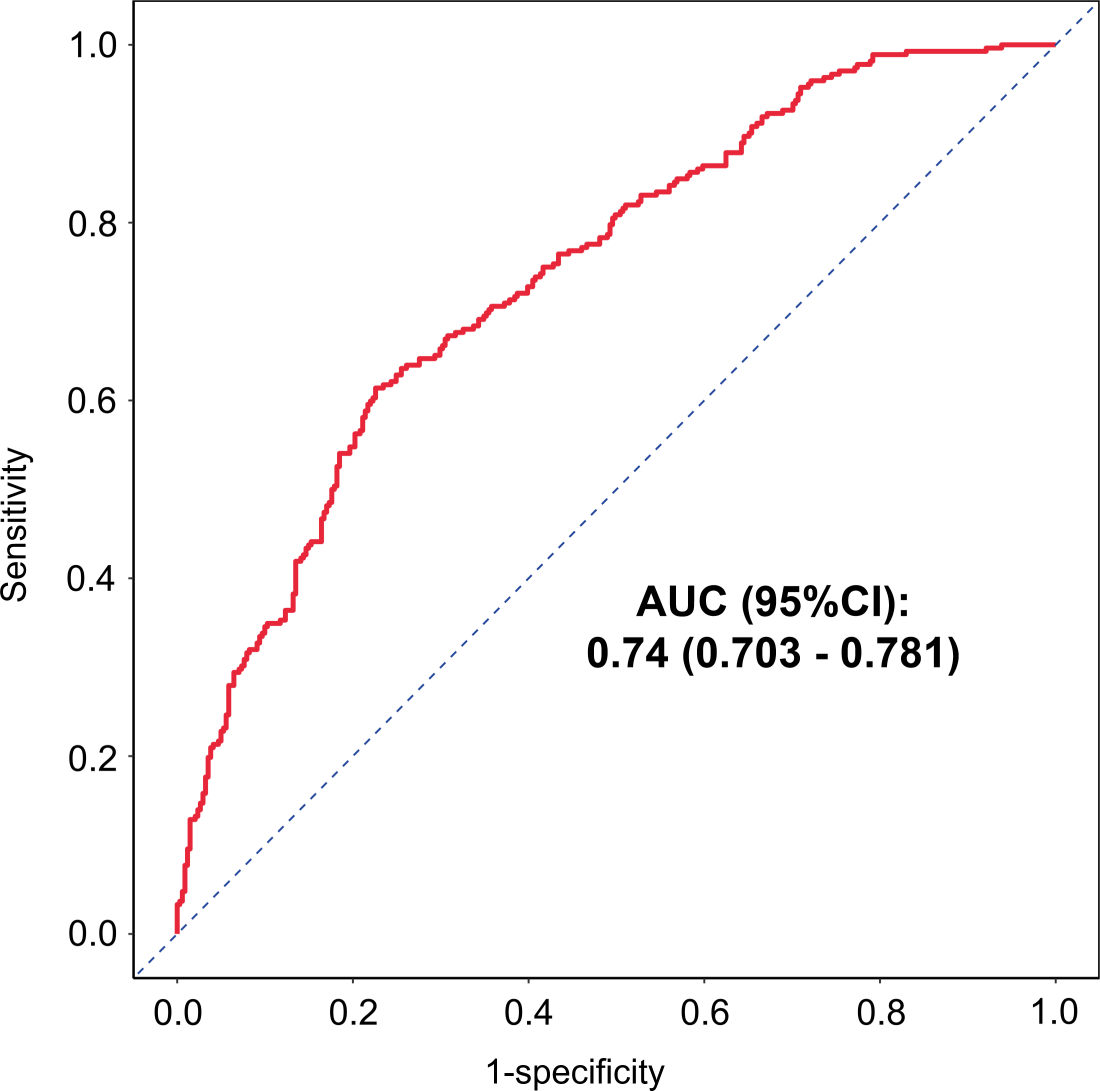
The ROC curve assessing the discriminative performance of the predictive model in advanced-age PCOS patients.

**Figure 7:**
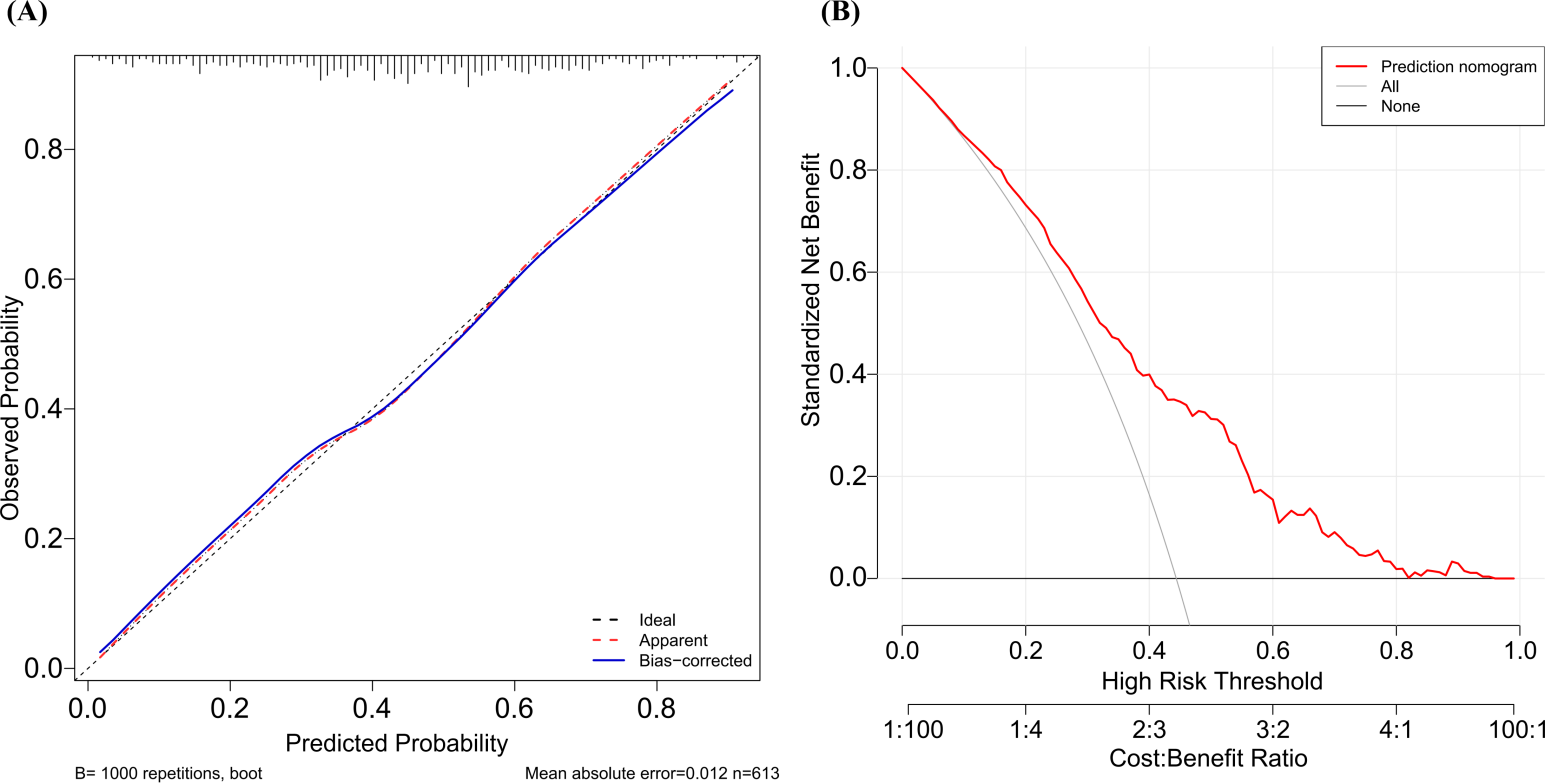
Evaluation of model calibration and clinical utility. **(A)** Calibration curve showing the agreement between predicted and observed CLBR probabilities. **(B)** Decision curve analysis (DCA) illustrating the clinical net benefit of the predictive model in advanced-age PCOS patients.

The nomogram was evaluated using several metrics. The AUC was 0.74 (95% CI: 0.703–0.781; **Figure 6**), indicating moderate discriminative ability. Internal validation via 1000 bootstrap resamples showed good agreement between predicted and observed probabilities on calibration curves (**Figure 7A**). The Hosmer-Lemeshow test yielded *P* = 0.108, supporting adequate model fit. Decision curve analysis demonstrated that the nomogram provided greater net clinical benefit compared with both the treat-all and treat-none strategies across a wide range of clinically reasonable threshold probabilities (**Figure 7B**).

## Discussion

This study demonstrated that among PCOS patients undergoing ART, CLBR remained comparable across age groups below 35 years but declined substantially in those aged 35 and older (44.40% vs. 63.90%–69.40%), even after adjusting for AFC and other covariates. Within the advanced-age subgroup, total Gn dose showed a non-linear association with CLBR, reaching a plateau at approximately 1, 600 IU. In contrast, EMT exhibited a stable positive linear association with CLBR throughout the measured range. These findings suggest that for advanced-age PCOS patients, clinical management may benefit from moderate stimulation combined with endometrial optimization rather than dose escalation alone.

Among PCOS patients, CLBR was preserved before age 35 but declined markedly in the ≥35 group. This threshold of 35 years mirrors the widely accepted definition of advanced maternal age in the general reproductive population, as stated in ACOG Obstetric Care Consensus No. 11 (21). Biological aging increases embryonic aneuploidy through disrupted meiotic spindle assembly and impaired mitochondrial function (22). In PCOS, the combination of hyperandrogenism, insulin resistance, and age-related changes may amplify oxidative stress (23) and chronic inflammation in the follicular environment (24), further reducing oocyte quality (25, 26). The age-related decline in CLBR observed in our cohort, despite preserved AFC, confirms that follicle quantity alone exhibits limited ability to predict reproductive success in this population.

Previous studies have reported a negative correlation between Gn dose and LBR, but those findings were based on linear assumptions. In our total cohort, Gn dose also showed a negative linear association with CLBR (aOR 0.979 per 100 IU). However, this linear model imposed a single average effect across all ages, potentially masking subgroup-specific dose-response patterns. When the analysis was restricted to the advanced-age subgroup, a non-linear pattern emerged: a positive association below approximately 1, 600 IU and a plateau above this threshold. This shift likely reflects confounding by indication and age-related effect modification. Given that Gn dosage was not randomized, dose escalations typically reflected clinical responses to suboptimal follicular growth. Across more than 650, 000 ART cycles, the negative dose-outcome association has been shown to flatten among women aged ≥35 years with few retrieved oocytes (27). The effect of FSH dosing on live birth rate also differs by age group, as demonstrated in a secondary analysis of the OPTIMIST trial (28). In predicted poor responders, increasing the starting dose from 225 to 375 IU/day did not improve live birth rates in a randomized trial (29). According to a Cochrane systematic review, the individualization of initial Gn dosages based on ovarian reserve markers does not yield superior ongoing pregnancy or live birth rates compared to standardized dosing regimens (30). These data collectively suggest that higher Gn exposure does not overcome the limitations imposed by ovarian aging. In ovaries of advanced reproductive age, the recruitable follicular cohort is inherently restricted. Below a total dose of approximately 1, 600 IU, escalation may still facilitate sufficient follicular recruitment to enhance the CLBR. Beyond this threshold, it appears that maximal recruitment has been achieved. Whether supra-threshold Gn exposure further compromises oocyte competence, potentially through mechanisms such as premature luteinization, disrupted cumulus-oocyte communication, or exacerbated oxidative stress, remains to be elucidated (31, 32). These effects cannot be definitively isolated from confounding by indication within the present dataset. Consequently, the 1, 600 IU threshold may serve as a marker of impending poor ovarian response rather than a definitive pharmacological ceiling. If this threshold primarily reflects a depleted ovarian reserve, dose reduction in isolation would likely be insufficient to improve clinical outcomes. This non-linear pattern is broadly consistent with observations in non-PCOS populations, where a threshold of approximately 1, 410 IU has been identified, beyond which further dose escalation no longer improves and may even reduce LBR (33). Similarly, daily Gn doses exceeding 450 IU or total doses above 3, 000 IU per cycle have been associated with significantly lower live birth rates (34). Consequently, in advanced-age PCOS patients, dose escalation beyond approximately 1, 600 IU may yield diminishing returns. This finding is preliminary and should not be used as a clinical predictor of CLBR without prospective validation.

In our cohort, the EMT was significantly lower in patients with advanced-age PCOS compared to their younger counterparts. Within the older subgroup, EMT showed a consistent positive association with CLBR. This finding was consistent with large-scale studies that identify a thin endometrium as a risk factor for lower LBR (35, 36). In PCOS, hyperandrogenism and insulin resistance impair estrogen-mediated endometrial proliferation. High androgen levels reduce the expression of endometrial receptivity markers, including IGFBP-1 and LIF, thereby directly compromising the implantation window (37). Chronic low-grade inflammation, a characteristic feature of PCOS, further disrupts the uterine immune environment through elevated pro-inflammatory cytokines, including IL-6 and TNF-α (38). These PCOS-specific changes are compounded by advanced age, which independently promotes the accumulation of senescent cells in the endometrial epithelium and reduces endometrial regenerative capacity (39, 40). In PCOS, thin endometrium is predominantly a downstream manifestation of systemic metabolic and hormonal dysregulation rather than an independent uterine defect. Hyperandrogenism, insulin resistance, and chronic low-grade inflammation impair endometrial proliferation and vascularization through disrupted PI3K/AKT and Wnt/β-catenin signaling (41). Notably, histomorphometric abnormalities persist despite progesterone supplementation and remain correlated with androgen and insulin levels (42). Given that EMT primarily reflects underlying metabolic dysfunction, interventions targeting the endometrium alone may not fully reverse the implantation defect. Nevertheless, partial improvement in endometrial thickness through targeted interventions may still confer clinical benefit. Accordingly, several approaches have been investigated in recent years. Prolonged estrogen supplementation and sildenafil are nowadays used to improve endometrial blood flow (43), while intrauterine vascular endothelial growth factor (VEGF) infusion promotes angiogenesis in animal models (44). Among emerging regenerative therapies, platelet-rich plasma (PRP) has shown the most robust evidence. A recent meta-analysis of randomized controlled trials reported that PRP significantly increased endometrial thickness and clinical pregnancy rate (45), and a network meta-analysis ranked PRP highest among endometrial interventions for clinical pregnancy rate (46). The linear association observed in the present study across the full EMT range (6–22 mm) suggests that incremental gains in endometrial thickness may be associated with higher live birth probability. Whether this association reflects a causal relationship or merely identifies patients with milder metabolic dysfunction requires further investigation.

Overall, these results highlight a practical consideration in managing older PCOS patients. Age-related decline and the metabolic features of PCOS may together compromise endometrial receptivity, yet clinicians may increase the Gn dose in an attempt to maximize oocyte yield. Our data suggest that dose escalation beyond approximately 1, 600 IU was not associated with higher CLBR in this cohort, although this observation requires prospective confirmation. Moderate stimulation protocols may achieve comparable outcomes while reducing medication costs. This is particularly relevant given that further dose escalation in patients with suboptimal follicular response imposes an economic burden without demonstrating an improvement in CLBR in the present data. Balancing stimulation intensity against endometrial preparation is therefore a relevant consideration in treatment planning for this population. To help quantify this trade-off, this study combined five readily available clinical variables into a prediction nomogram. The model showed acceptable discrimination (AUC = 0.74) and good calibration, and decision curve analysis confirmed a net clinical benefit across a broad range of threshold probabilities. This tool may assist clinicians in estimating the probability of live birth and informing pre-treatment counseling, although external validation is needed before clinical adoption.

Several limitations of this study should be acknowledged. First, the total Gn dose was not randomly assigned but determined by physician judgment, introducing confounding by indication. The 1, 600 IU threshold may therefore partly reflect the clinical selection of a subgroup with poorer ovarian responsiveness rather than a pharmacological ceiling. It is still too early to conclude that the Gn dose-CLBR relationship is definitively non-linear in this population. Second, the threshold was derived from a single-center study using a GnRH antagonist protocol in Han Chinese women and requires external validation. Third, AMH was not routinely measured, which may have resulted in residual confounding by unmeasured ovarian reserve. Fourth, the nomogram has not been externally validated. Fifth, this study did not examine the direct association between Gn dose and EMT, and it remains unclear whether Gn dose influences CLBR, in part, through endometrial effects. Sixth, PCOS was diagnosed using the Rotterdam criteria without further phenotype stratification, which may have masked heterogeneity in treatment response across PCOS subtypes.

Future studies should validate the 1, 600 IU threshold in independent multi-center prospective cohorts with randomized dose allocation and comprehensive ovarian reserve assessment including AMH. Whether endometrial thickness-improving interventions can increase CLBR, and whether Gn dose influences CLBR in part through endometrial effects, also warrants further investigation.

## Conclusions

This study demonstrated that CLBR was significantly lower in PCOS patients aged ≥35 years compared with younger cohorts. A non-linear association between total Gn dose and CLBR was observed in the advanced-age subgroup, with a plateau at approximately 1, 600 IU. Given the retrospective design, this threshold likely reflects confounding by indication and should be interpreted with caution. A stable positive association between EMT and CLBR was identified. A predictive nomogram was constructed using five clinical variables. These findings suggest that moderate stimulation protocols with attention to endometrial preparation may warrant consideration in this population and help reduce medication costs.

## Data Availability

The original contributions presented in the study are included in the article/supplementary material. Further inquiries can be directed to the corresponding author.
